# Mapping QTL Associated with Photoperiod Sensitivity and Assessing the Importance of QTL×Environment Interaction for Flowering Time in Maize

**DOI:** 10.1371/journal.pone.0014068

**Published:** 2010-11-19

**Authors:** Cuiling Wang, Yanhui Chen, Lixia Ku, Tiegu Wang, Zhaohui Sun, Fangfang Cheng, Liancheng Wu

**Affiliations:** 1 College of Agronomy, Henan Agricultural University, Zhengzhou, People's Republic of China; 2 College of Agronomy, Henan University of Science and Technology, Luoyang, People's Republic of China; 3 College of Life Science, Henan Institute of Science and Technology, Xinxiang, People's Republic of China; 4 Gongyi Meteorological Office, Gongyi, People's Republic of China; University of Umeå, Sweden

## Abstract

**Background:**

An understanding of the genetic determinism of photoperiod response of flowering is a prerequisite for the successful exchange of germplasm across different latitudes. In order to contribute to resolve the genetic basis of photoperiod sensitivity in maize, a set of 201 recombinant inbred lines (RIL), derived from a temperate and tropical inbred line cross were evaluated in 5 field trials spread in short- and long-day environments.

**Methodology/Principal Findings:**

Firstly, QTL analyses for flowering time and photoperiod sensitivity in maize were conducted in individual photoperiod environments separately, and then, the total genetic effect was partitioned into additive effect (A) and additive-by-environment interaction effect (AE) by using a mixed-model-based composite interval mapping (MCIM) method.

**Conclusions/Significance:**

Seven putative QTL were found associated with DPS thermal time based on the data estimated in individual environments. Nine putative QTL were found associated with DPS thermal time across environments and six of them showed significant QTL×enviroment (QE) interactions. Three QTL for photoperiod sensitivity were identified on chromosome 4, 9 and 10, which had the similar position to QTL for DPS thermal time in the two long-day environment. The major photoperiod sensitive loci *qDPS10* responded to both short and long-day photoperiod environments and had opposite effects in different photoperiod environment. The QTL qDPS3, which had the greatest additive effect exclusively in the short-day environment, were photoperiod independent and should be classified in autonomous promotion pathway.

## Introduction

Flowering time is known to be an important reproductive characteristic of agronomic interest and plays a principal role in the geographical adaptability of plants, the expression of which highly depends on environmental conditions. Photoperiod is one of the most important environmental signals that determine when a plant will flower. Photoperiod sensitivity can be considered to be a survival characteristic because it provides a safety mechanism ensuring that crop reproduction will occur under favorable environmental conditions. Photoperiod sensitivity also results in plants not adapting to environments outside the ecogeographical ranges of their wild ancestors and is the major obstacle of exchange between different geographical regions. Being a short-day plant, the flowering time of maize is promoted by short photoperiods, i.e. the longer the period of day length is, the later maize plants flower. Most tropical maize germplasm show significant photoperiod sensitivity and delayed flowering in temperate zone, some tropical varieties even do not flower under temperate environmental regions [1]–[3]. An understanding of the genetic determinism of flowering time in different photoperiod environment is a prerequisite for the usage of tropical maize germplasm in temperate areas.

Previous studies have proposed that the photoperiod critical threshold is 12 to 13 hours, beyond that period, the thermal time necessary for the photoperiod sensitive maize germplasm to flower increases linearly as day length increases [4]–[7]. Results from classical genetic research and breeding programs demonstrate that, like many other important traits in plant breeding, flowering time and photoperiod sensitivity are complex traits that show continuous phenotypic variation among progeny and are controlled by multiple genes, and to a large extent, controlled by additive genes [2], [5], [8]–[10]. Previous studies show that flowering time in maize is determined by two components: base maturity and photoperiod sensitivity, and the two components are governed by separate genetic mechanisms [8], [11]–[12].

With the availability of molecular markers to develop well-saturated genetic maps, mapping quantitative trait loci (QTL) has become a standard procedure to study the genetic architecture of quantitative traits, because it allows the estimation of the QTL number, their genomic position, and the genetic effects of the QTL that control them. A large body of QTL data for flowering time, associated with different environmental parameters is presently available [12]–[23]. Chardon et al. employed a metadata-analysis methodology on 312 publicly available QTL for flowering time and generated a synthetic genetic model with 62 consensuses QTL, and determined that hot-spot loci were located on chromosomes 1, 8, 9 and 10 [24]. Using a set of 5000 recombinant inbred lines of a maize nested association mapping population, Buckler at el. demonstrated the genetic architecture of flowering time was not caused by a few genes of large effect, but by the cumulative effects of numerous small additive QTLs with few genetic or environmental interactions [25]. However, few QTL resolved in previous studies directly addressed photoperiod sensitivity and photoperiod sensitivity was not considered to be a major factor segregating in most cross. Therefore, little is known about the genetic base of flowering by photoperiod in maize and the knowledge on the chromosome region, structure and function of photoperiod sensitivity genes in maize is relatively poor.

Many important questions regarding the genetic aspects of photoperiodic response of flowering remain largely unanswered. These include the following: How frequently do flowering time QTL interact with environments? Which QTL are independent of photoperiod and which are involved in photoperiod response? Consequently, there is a need to understand more about the ways in which flowering time is affected by photoperiod at the molecular level in maize.

In order to establish the genetic basis of maize photoperiod sensitivity, Koester et al. identified flowering time, plant height and leaf number QTL under different photoperiod environments and speculated that maturity QTL on chromosome 8 may represent a photoperiod response element [12]. Moutiq et al. compared flowering time QTL in different photoperiod environments, and suggested that QTL on chromosomes 8 and 10 had the greatest additive effects during long days, while those on chromosomes 3 and 9 had increased additive effects during short days [9]. Wang et al. found that QTL for flowering time, plant height and leaf number, under long-day conditions, were clustered on chromosome 10, while QTL for short day conditions resided on chromosome 3. The QTL in the bin 10.04 region of chromosome 10 were detected associated with photoperiod sensitivity and related traits during long-days [26]. Previous studies of photoperiod sensitivity were either undertaken by comparison of flowering time QTL identified in different photoperiod environments to indirectly speculate the photoperiod sensitivity QTL or by introducing an index such as PPR and PS to directly evaluate and identified QTL for photoperiod sensitivity in maize, or with the two previous designs [9], [26]. QTL analysis of photoperiod sensitivity has generally shown that different QTL for flowering time were detected in different photoperiod environments on the same population, which indicated that there are high level of QTL×environment interaction for all those loci. Most commonly, a major photoperiod sensitivity QTL is reported when a QTL is detected having great effect only in long-day environment and not in short-day environment in most previous study. Yet this is not a statistical test, and can be misleading if a QTL is present but at just below the operational significance threshold in one environment and above it in another.

Buckler et al. reported that no individual QTLs associated with flowering time were determined by geographic origin or large effects for epistasis or environmental interactions [25]. However, the testing environments of Buckler et al. differenced in temperatures and rainfall, and day lengths were consistently longer than the critical photoperiod for short-day maize. Therefore, up to now, there have been no reports that precisely evaluated the interaction between flowering time QTL and photoperiod sensitivity environment, as well as successfully distinguished base maturity QTL and photoperiod sensitivity QTL from flowering time QTL, due to the lack of appreciated analysis method and experiment design.

The software of QTLNetwork version 2.0 based on the mixed linear model has been developed for mapping QTL with additive effects and epistatic effects as well as their QE [27]–[28]. This method has been successfully applied in a number of recent QTL mapping studies [29]–[31]. In this paper, we present a novel genetic investigation of photoperiod response in maize in three photoperiod environments, using a mixed-model-based composite interval mapping (MCIM) method, based on a recombinant inbred line (RIL) population derived from a temperate×tropical cross.

The objective of this study was to (1) identify the QTL associated with flowering time under different photoperiod environments; (2) test QTL×E interaction ; and (3) characterize photoperiod sensitivity QTL.

## Materials and Methods

### Plant material and field trials

A RILs population consisted of 201 F_10_ recombination inbred lines, derived from a cross between two inbred lines, Huangzao4 and CML288, using a single-seed descent method under short-day conditions (Sanya, China, 18°45′N, 109°30′E). The parent, Huangzao4, is a temperate photoperiod insensitive inbred line derived from a local Chinese germplasm, Tangsipingtou, a heterotic group used broadly in China. CML288 is a tropical photoperiod sensitive flint inbred line introduced from CIMMYT.

### Field evaluation

Evaluation of the RILs population, two parents and F_1_ was conducted in the field in 2007 under a short day environment of Sanya(18°45′N, 109°30′E), long-day environments of Zhengzhou (34°43′N, 113°43′E)and Luoyang(34°39′N, 112°28′E ) in Henan province, and long-day environments of Shunyi(40°07′N, 116°39′E) and Changping(40°14′N, 116°13′E) in Beijing(Table 1). The field experiment was designed according to a complete randomized block design with three replications at each location. Each RIL was planted in one row, 0.67m apart and 4m long with a total of 15 plants per row; the density was 45000 plant/ha. Field management was in accordance with local practices.

**Table 1:** Locations and environmental characteristics of five field trails.

					Temperature(°C)		
Location	Longitude	Latitude	Sowing date	Daylength(h)^a^	highest	lowest	mean
Sanya	109°30′E	18°45′N	Mar 19	12.4	29.5	23.8	27.2
Zhengzhou	113°43′E	34°43′N	April 26	14.3	31.4	17.4	25.5
Luoyang	112°28′E	34°39′N	May 2	14.3	30.8	16.1	25.4
Changping	116°13′E	40°14′N	May 16	14.8	30.7	17.6	25.3
Shunyi	116°39′E	40°07′N	May22	14.8	30.4	18.6	25.7

Maize is sensitive to photoperiod at the stage of tassel initiation [4]. Considering the slow rate of change of photoperiod, the photoperiod during tassel initiation was assumed as the photoperiod of sensitive stage in each environment. However, tassel initiation could not be determined directly, in this study, it was estimated as half the average thermal time necessary from sowing to silking according to the method of Bonhomme et al. and Gouesnard et al. [3], [6]. The average photoperiod of sensitive stage is 12.4h at Sanya short-day environment of, 14.3h at Henan long-day environment and 14.8h at Beijing long-day environment.

### Data collection

Maize is a monoecious plant and its flowering involves two progress, male flowering and female flowering. Considering female flowering may indeed be delayed because of diseases and other abiotic stress and, in this study, many lines even can not silk in long day environment due to photoperiod sensitivity, only male flowering was used for the estimation of flowering time and photoperiod sensitivity. Traits were measured from ten consecutive plants beginning with the third plant of each row. Days to pollen shed (DPS) were recorded as the number of days from sowing to the first pollen shed from anthers on the central spike. The arithmetic mean values for DPS were subsequently transformed into thermal time, and used to detect flowering time QTL. Thermal time (TT) was calculated as:
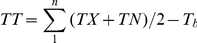
where TT is the thermal time accumulated over n days, TX is the maximum daily temperature, TN is the minimum daily temperature, and Tb is the base temperature [3], [6]. For all locations, Tb was set at 10°C. Photoperiod sensitivity was evaluated in Henan and Beijing environment, respectively. Photoperiod sensitivity (PS) was calculated as: PS = (TT_LD_−TT_SD_)/(DL_LD_−DL_SD_), where TT_SD_ is the thermal time of each RIL from sowing to days to pollen shed in short-days of Sanya, TT_LD_ is the thermal time of each RIL from sowing to days to pollen shed in long-days, DL_SD_ is the average day length of photoperiod sensitivity stage in short-day environment, DL_LD_ is the average day length of photoperiod sensitivity stage in long-day environment (PS°C/h) [6]. The data obtained from ten consecutive plants were averaged to obtain trait values for each plot, and three replications were averaged to obtain trait values for each line in each experiment. To increase the veracity of the evaluation, the arithmetic mean values of each line at Zhengzhou and Luoyang location were averaged to obtain RIL trait values in long day environment of Henan province, and the arithmetic mean values of each line at Shunyi and Changping location were averaged to obtain RIL trait values in long day environment of Beijing.

Broad-sense heritability (*h^2^*) for flowering time in each environment was computed according to Knapp [32]. The heritability was calculated as follows: *h^2^* = *σ_g_^2^*/(*σ_g_^2^*+*σ_gl_^2^/n*+*σ_e_^2^/nr*), where *σ_g_^2^* is the genetic variance, *σ_gl_^2^* is genotype-by-location interaction (where location means locations within a single photoperiod environment experiment rather than locations in all photoperiod environment experiment), *σ_e_^2^* is the error variance, *r* is the replication number, and *n* is the number of location in a single photoperiod environment. The estimates of *σ_g_^2^*, *σ_gy_^2^*, and *σ_e_^2^* were obtained by analysis of variance (ANOVA) using the general linear model procedure of the statistical software SPSS 12.0.

### Molecular linkage construction and QTL mapping

In accordance with bin location, a total of 713 SSR markers were chosen from the maize genome database to detect parental polymorphisms,according to the protocol available at http://www.maizegdb.org/documentation/maizemap/ssr_protocols.php, with minor modifications. The co-dominant segregation SSR markers were used to genotype the RIL population. The genetic linkage map was constructed with Mapmaker/Exp 3.0 at the LOD threshold >3.0 [33].

QTL analyses were performed using mixed linear composite interval mapping in the software QTLNetwork 2.0, based on a mixed linear model [27], [28]. Testing window, work speed and filtration window set at 10 cM, 2 cM and 10 cM, respectively. Significance testing was based on the F-test using Henderson method III, and 10,000 permutation tests were used to calculate the critical F-value to control the genomewise type I error [34], [35]. QTL detection was undertaken for each environment separately at first and then across environments. QTL identified in individual environment were expected to contain mixed effects of additive effect (A) and additive-by-environment interaction effect (AE), while QTLs obtained across environments were with A and AE, respectively.

## Results

### Phenotypic measurement of photoperiod sensitivity and flowering time in different photoperiod environments

**Figure 1:**
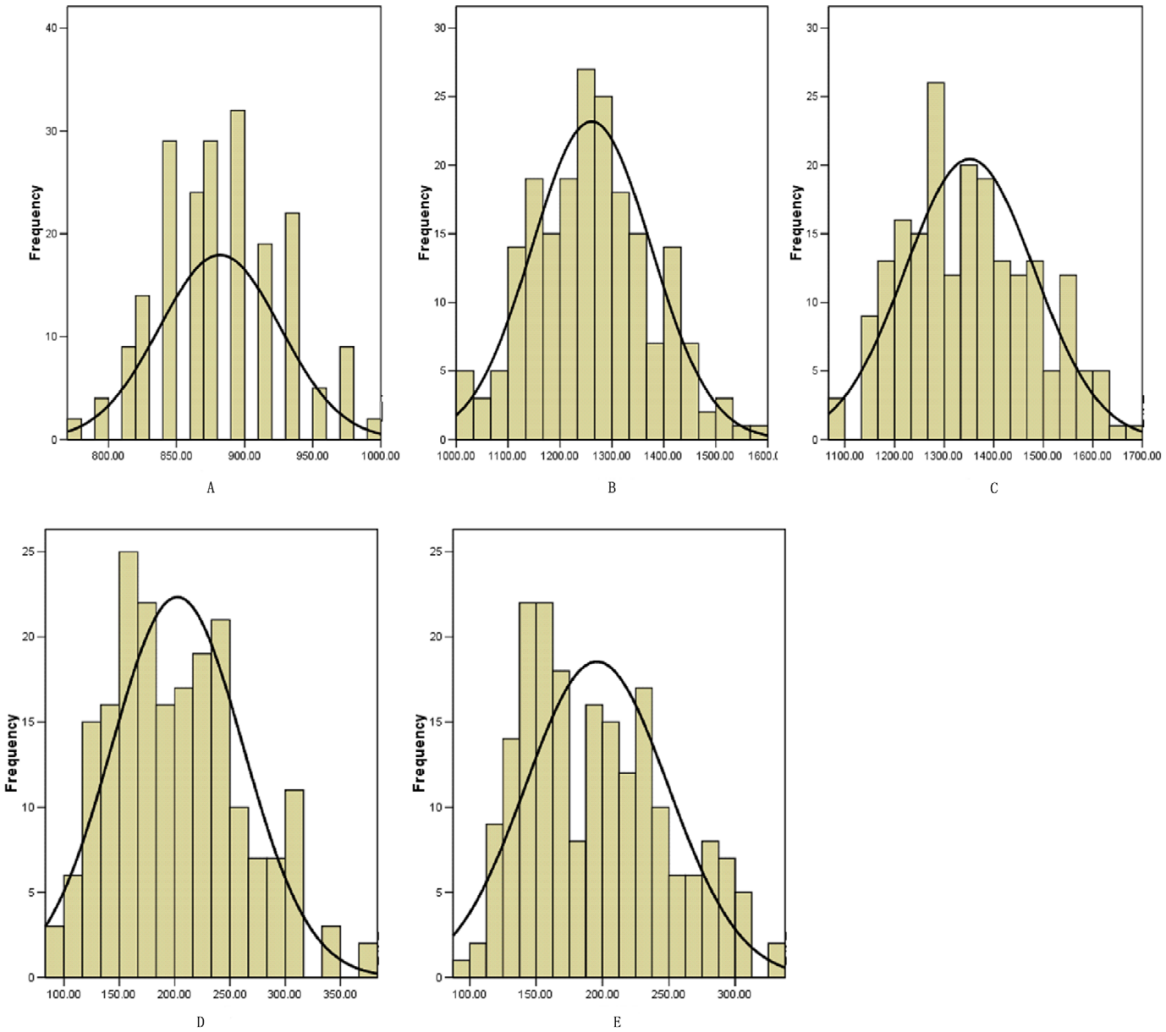
Frequency distribution for flowering time and photoperiod sensitivity in the recombinant inbred lines(RILs) derived from the cross Huangzao4×CML288. (A) flowering time in Sanya (B) flowering time in Henan (C) flowering time in Beijing (D) photoperiod sensitivity in Henan (E) photoperiod sensitivity in Beijing.

Huangzao4 and CML288 were significantly different for flowering time in all the three photoperiod environments (P<0.01). Thermal time from sowing to days to pollen shed for both parents were significantly increased along with the increase of latitude and CML288 showed more strong photoperiod sensitivity than Huangzao4 (Table 2). Thermal time from sowing to days to pollen shed for CML288 grown in the long day environments of Henan and Beijing increased by 61.74% and 86.23%, respectively, compared with grown in short day conditions of Sanya, while Huangzao4 increased by only 24.02% and 35.81%, respectively. What's more, CML288 did not silk in Henan and Beijing environment over the study period. Photoperiod sensitivity values of CML288 were approximately three times that observed in Huangzao4, which indicated that CML288 exhibited increased photoperiod sensitivity. In all environments, the flowering time and photoperiod sensitivity measured in the RIL population followed approximately a normal distribution presenting a suitable phenotypic segregation for QTL mapping(Fig. 1). Compared with RILs grown in low latitude area in Sanya, RILs grown in high latitude in Henan and Beijing showed 38.12% and 60.43% increase in thermal time of flowering time (DTT) respectively. Transgressive segregation in both directions was observed for flowering time under the three photoperiod environments and for photoperiod sensitivity under long day environments.

**Table 2:** Phenotypic evaluation of the two parents, F_1_ and the RIL population in three environments.

	traits	TT^a^			PS^a^	
		Sanya(18°45′N)	Henan(34°39′N–34°43′N)	Beijing(40°07′N∼40°14′N)	Henan(34°39′N–34°43′N)	Beijing(40°07′N∼40°14′N)
Huangzao4(*P_1_*)	Mean	823.38	1021.12	1118.24	105.74	122.86
CML288(*P_2_*)	Mean	958.67	1550.52	1785.33	316.5	344.44
*P_1_* VS *P_2_* b		**	**	**	**	**
F_1_	Mean	783.43	1136.88	1264.47	189.01	200.43
RIL	Mean±sd	881.83±44.52	1218.04±107.47	1414.71±137.03	225.64±69.77	297.7±75.53
	Range	777.2–990.3	976.48–1508.06	1118.4–1746.8	89.08–431.64	144.08–487.49
	Skewness	0.18	0.21	0.34	0.45	0.46
	Kurtosis	−0.41	−0.14	−0.51	−0.33	−0.49
	H_B_^2^ c(%)	82.82	85.56	93.04	86.35	82.67

Note:

a , TT: flowering time, estimated as the sum of effective temperature from sowing to days to pollen shed;

PS: photoperiod sensitivity, calculated as: PS = (TTLD−TTSD)/(DLLD−DLSD), where TTSD is the thermal time of each RIL from sowing to days to pollen shed in short-days of Sanya, TTLD is the thermal time of each RIL from sowing to days to pollen shed in long-days, DLSD is the average day length of photoperiod sensitivity stage in short-day environment, DLLD is the average day length of photoperiod sensitivity stage in long-day environment.

b , Statistical test for difference between two parents at 0.05 (*) and 0.01 (**) levels of probability; ns, not significant.

c ,H_B_^2^, the broad-sense heritability.

Variance analysis was conducted using the mixed model. Difference between repetitions was not significant while entries and the interaction of entry×environment were highly significant for flowering time and photoperiod. Broad sense heritability was calculated considering different data sets. When calculated using the raw data collected from each of location of each photoperiod environment, heritability for flowering time was higher in Henan and Beijing long photoperiod environment (85.56% and 93.04%) and lower in short day environment (82.82%), when calculated across environments, heritability for flowering time was reduced (76.14%).

### Genetic linkage map construction

A total of 713 SSR markers were used to screen polymorphisms between the two parental inbred lines. Two hundred seventy-nine distinct co-dominant markers were employed to construct a genetic linkage map. Two hundred thirty-seven informative markers were assigned to 10 chromosomes using Mapmaker 3.0 at LOD>3.0. The linkage map had a total length of 1974.3 cM with an average interval of 8.33 cM between adjacent makers.

### QTL detection for flowering time in different photoperiod environments

#### QTLs with A+AE effects on DPS thermal time

**Figure 2:**
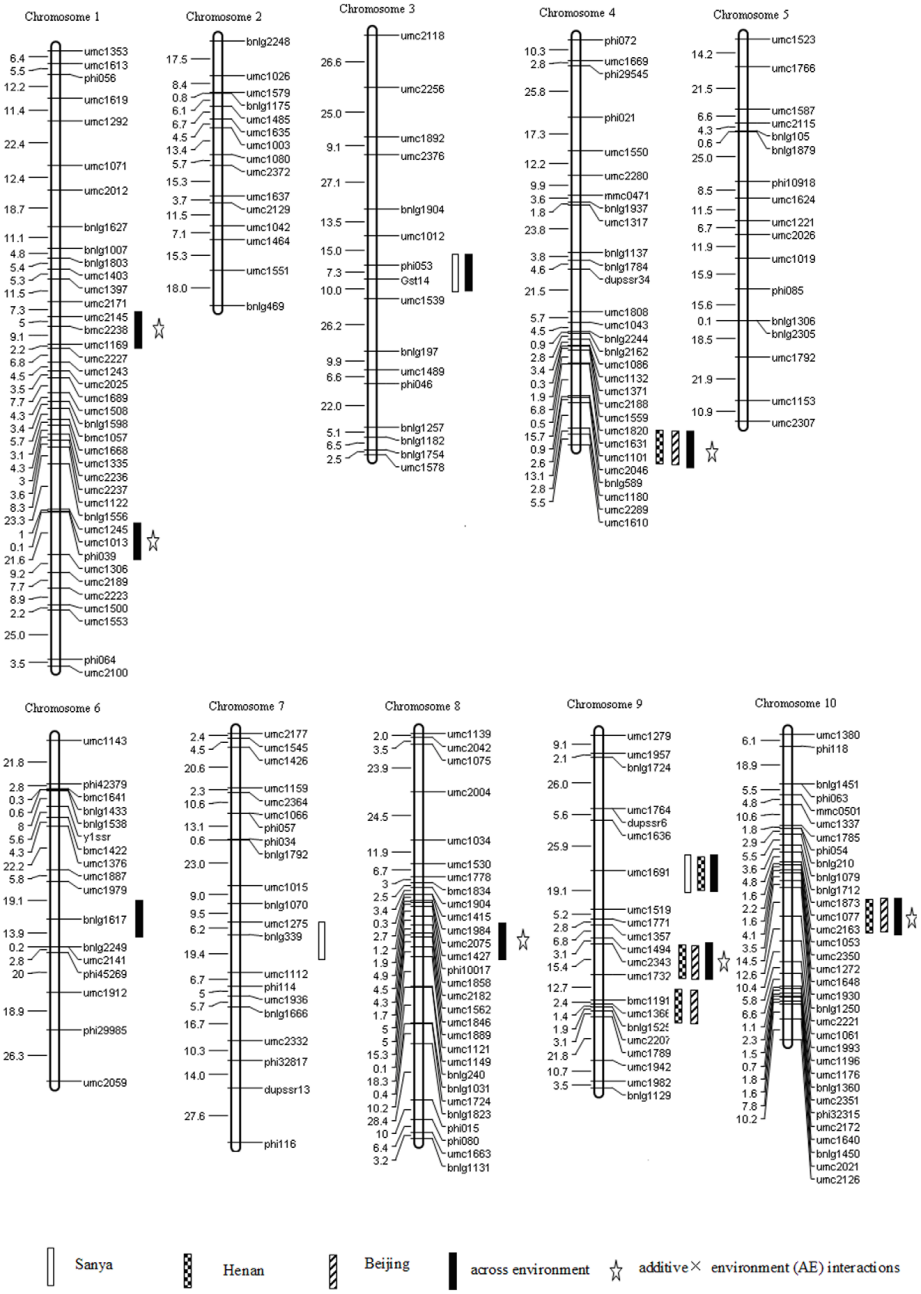
The position of major QTL and AE interaction detected for flowering time in three photoperiod environments.

QTL mapping based on the data estimated in individual photoperiod environments led to the identification of the QTL with mixed effects of additive effect (A) and additive-by-environment interaction effect (AE) for flowering time in the RILs in maize. In total, seven putative QTL with mixed effects of A+AE were found associated with DPS thermal time, which were mapped to chromosome 3, 4, 7, 9 and 10 (Table 3 and Fig. 2). The phenotypic variance explained by the QTL ranged from 3.45 to 44.30% and the detected QTL totally explained 34.89%, 72.12% and 65.76% of the phenotypic variance in Sanya, Henan and Beijing, respectively. Trait values at all detected QTL were increased from the allelic contributions of CML288. Among these QTL, none of the same QTL was detected simultaneity under all the three photoperiod conditions, however, four (*qDPS4*, *qDPS9-2*, *qDPS9-3* and *qDPS10*) were identified in both Henan and Beijing environments, whereas were not detected in Sanya environment; one (*qDPS9-1*) were identified in both Henan and Sanya environments; two (*qDPS3* and *qDPS7*) were identified only in Sanya environment. The QTL, *qDPS10* located on chromosome 10.04 between markers umc1873-umc2163, demonstrated the highest additive effects with values of 75.08°C–95.49°C and accounted for 39.79%–44.30% of the phenotypic variance for DPS thermal time in Henan and Beijing long-day environment. The QTL, *qDPS3* located on chromosome 3.05 between markers phi053-umc1539, demonstrated the highest additive effects with values of 25.62°C and accounted for 27.16% of the phenotypic variance for DPS thermal time in Sanya short-day environment. No significant epistasis interaction associated with DPS thermal time was detected.

**Table 3:** The QTL detected for thermal time for flowering time in individual photoperiod environments.

Environment	QTL	Cloest marker	Position	Support interval	A^a^	SE	h∧2(a)^b^
Sanya (short day)	*qDPS3*	Gst14	128.3	125.3–132.6	−25.62	2.62	0.2716
	*qDPS7*	bnlg339	99.6	89.1–113.8	−10.81	2.77	0.0375
	*qDPS9-1*	umc1691	63.8	49.8–73.7	−13.14	3.04	0.0398
Henan(long day)	*qDPS4*	umc1631	170.2	163.7–177.2	−25.36	6.04	0.0389
	*qDPS9-1*	umc1691	57.8	43.8–77.7	−27.74	6.46	0.0345
	*qDPS9-2*	umc2343	110.7	105.6–115.7	−21.46	5.80	0.1074
	*qDPS9-3*	bmc1191	130.1	125.1–134.8	−23.96	5.63	0.1425
	*qDPS10*	umc1077	62.7	60.7–64.5	−75.08	5.20	0.3979
Beijing (long day)	*qDPS4*	umc1820	160.9	156.9–175.2	−29.91	7.01	0.0389
	*qDPS9-2*	umc2343	111.7	105.6–118.7	−27.22	7.55	0.0728
	*qDPS9-3*	bmc1191	129.1	123.1–134.8	−26.14	7.34	0.1029
	*qDPS10*	umc1077	62.7	60.7–64.5	−95.49	6.65	0.443

Note:

a : Additive effect: positive values indicated that Huangzao4 carries the allele for an increase in the trait, while negative values indicate that CML288 contributed the allele for an increase in the trait value.

b , Contribution explained by putative main-effect QTL.

#### QTLs with A effects on DPS thermal time

Quantitative trait locus mapping based on the data estimated across environments led to the identification of the QTL with small but stable effects in different environments (Table 4). In totally, 9 putative QTL were found associated with DPS thermal time across environments. These QTLs were located on 7 maize chromosomes: two on chromosome 1 and chromosome 9 and one on each of chromosomes 3, 4, 6, 8 and 10. These QTLs explained from 0.1% to 4.4% of the phenotypic variance with the additive effects ranging from 13.05°C to 55.04°C. The *qDPS10* had the most significant effect, accounting for 4.45% of the phenotypic variance. Trait values at all detected QTL were increased from the allelic contributions of CML288. The total additive QTL detected for maize flowering time accounted for 8.05% of the phenotypic variance. Some QTL (*qDPS1-1*, *qDPS1-2*, *qDPS6* and *qDPS8*) were detected across environments but not in individual environment, while *qDPS7* and *qDPS9-3* were detected in individual environment but not across environments.

**Table 4:** Estimated additive (A) and additive×environment (AE) interactions of QTLs for flowering time in three photoperiod environments.

QTL	Cloest marker	position	Support interval	A^a^	AE1^b^	AE2^b^	AE3^b^	h∧2(a)^c^	h∧2(ae)^d^
*qDPS1-1*	umc2238	137.4	135.4–140.4	−13.05**	9.06*			0.0038	0.0014
*qDPS1-2*	umc1013	231.2	223.9–232.3	−14.48**	8.16*			0.0021	0.0010
*qDPS3*	Gst14	130.6	123.3–137.6	−16.09**				0.0038	
*qDPS4*	umc1631	169.2	164.2–173.2	−23.68**	17.55**		−13.23**	0.0072	0.0024
*qDPS6*	bnlg1617	83.4	72.4–90.5	−16.34**				0.0031	
*qDPS8*	umc2075	83.7	79.0–85.4	−17.57**	12.86**		−12.33**	0.0084	0.0036
*qDPS9-1*	umc1691	60.8	49.8–75.7	−16.48**				0.0010	
*qDPS9-2*	umc2343	110.7	106.7–114.7	−23.36**	19.83**		−11.48*	0.0066	0.0025
*qDPS10*	umc1077	62.7	61.7–63.7	−55.04**	58.56**	−18.48**	−40.40**	0.0445	0.0256

Note:

a : Additive effect: positive values indicated that Huangzao4 carries the allele for an increase in the trait, while negative values indicate that CML288 contributed the allele for an increase in the trait value.

b , additive×environment (AE) interactions effect:E1, Sanya; E2, Henan; E3, Beijing.

c , Contribution explained by putative main-effect QTL.

d , Contribution explained by additive×environment (AE) interactions.

#### QTLs with AE interaction effects on DPS thermal time

Six QTL were involved in significant AE interactions for DPS thermal time (Table 4): two on chromosome 1 and one on each of chromosomes 4, 8, 9 and 10, respectively. The AE effects explained from 0.1% to 2.56% of the phenotypic variance. *qDPS1-1* and *qDPS1-2* showed significant ae interaction effect only in Sanya short day environments, *qDPS4*, *qDPS8* and *qDPS9-2* showed significant ae interaction effect both in Sanya short day environments and in Beijing long day environment, however, differences were observed in both the magnitudes and directions of effects. *qDPS10* showed the largest variation among environments, with ae values of 58.56, −18.48 and −40.40 in Sanya, Henan and Beijing, respectively.

### QTL detection for photoperiod sensitivity in maize

The ANOVA (not shown) indicated that the interaction between lines and environment for photoperiod sensitivity was not significant. For this reason and for the sake of conciseness, the QTL detection was carried out for photoperiod sensitivity on the average performance of Henan and Beijing long day environment and within each environment. For photoperiod sensitivity, three QTL were identified on chromosome 4, 9 and 10. The phenotypic variance explained by the QTL ranged from 3.90 to 52.93%. Trait values at all detected QTL were increased from the allelic contributions of CML288. All QTL detected for photoperiod sensitivity had the similar position to QTL for DPS thermal time in the Henan and Beijing long-day environment (Fig. 2 and Table 5). No significant epistasis interaction associated with photoperiod sensitivity was detected.

**Table 5:** The QTL detected for photoperiod sensitivity in RIL population.

Environment	QTL	Cloest marker	Position	Support interval	A^a^	SE	h∧2(a)^b^
Henan	*qPS9*	umc2343	104.6	89.8–109.7	−22.49	3.46	0.0789
	*qPS10*	umc1077	62.7	60.7–64.5	−52.28	3.47	0.5003
Beijing	*qPS4*	umc1820	160.9	156.9–169.2	−16.14	3.88	0.0436
	*qPS9*	umc2343	107.7	102.6–116.7	−19.25	3.76	0.0399
	*qPS10*	umc1077	62.7	60.7–64.5	−57.05	3.68	0.5123
average	*qPS4*	umc1820	160.9	155.0–172.2	−14.15	3.55	0.039
	*qPS9*	umc2343	106.7	102.6–114.7	−20.03	3.32	0.0529
	*qPS10*	umc1077	62.7	61.7–64.5	−54.85	3.37	0.5293

Note:

a : Additive effect: positive values indicated that Huangzao4 carries the allele for an increase in the trait, while negative values indicate that CML288 contributed the allele for an increase in the trait value.

b , Contribution explained by putative main-effect QTL.

## Discussion

Up to now, the genetic basis of flowering time control has not been well understood in maize. In previous studies, QTL associated with photoperiod sensitivity has been inferred indirectly by comparing mapping results of flowering time QTL in different photoperiod environments and it was speculated that those QTLs detected only in long day environment might contain important photoperiod response element [9], [12], [26]. However, in any specific environment, the total effect of a QTL includes the main effect of the QTL and QE interaction effects for that environment. Therefore, conclusions of which QTL associated with photoperiod sensitivity by comparing mapping results of flowering time QTL in different photoperiod environments could only be speculative. Understanding how interactions between QTL and photoperiod environment and distinguishing which QTL involved in base maturity and which QTL in photoperiod sensitivity would be a step forward for understanding the genetic basis of flowering time and photoperiod sensitivity in maize. More recently, Buckler et al. demonstrated the genetic architecture of flowering time was not caused by a few genes of large effect, but by the cumulative effects of numerous small additive QTLs with few genetic or environmental interactions [25]. However, day lengths of their testing environments were consistently longer than the critical photoperiod for short-day maize. In this study, using a RIL population derived from a tropical×temperate cross, flowering time were evaluated in three different photoperiod environment (Sanya, Henan and Beijing) and the genetics of flowering time in maize were dissected into QTL with main effects and their interactions with photoperiod environments rigorously.

Based on this study, flowering time QTL might be divided into two classes: (1) base maturity QTL, i.e. autonomous promotion pathway QTL, having no interactions with photoperiod environment; and (2) photoperiod sensitivity QTL, having significant interaction effect with photoperiod environment. In this study, *qDPS3*, *qDPS6* and *qDPS9-1* were found only having main additive effect for flowering time and no interaction with any photoperiod environment, meaning that the three QTL should be classified in autonomous promotion pathway. Six QTL showed significant QE interactions for DPS thermal time (Table 4): two of them (*qDPS1-1* and *qDPS1-2*) showed significant QE interaction effect only in Sanya short day environments and the residual four QTL (*qDPS4*, *qDPS8*, *qDPS9-2* and *qDPS10*) showed significant QE interaction effect both in short day and long day environments. These six QTL respond to photoperiod environment and should be involved in photoperiod pathway.

The region of chromosome 3.05 has been detected associated with flowering time in many studies [9], [13], [14], [19], [26], [36], [37], [38]. In this study, based on the data estimated in individual photoperiod environment, mapping results showed that the major QTL for DPS thermal time, qDPS3, located in the bin of 3.05 on the chromosome 3 had the greatest additive effect exclusively in the short-day environment. Moutiq et al. [9] also reported that qDPS3 only detected in short-day environment but not in long-days. It should be pointed that, based on the data estimated across environments, the QTL qDPS3, were found have no interaction with photoperiod environment, meaning that this QTL were photoperiod independent and should belong to base maturity rather than‘photoperiod promotion pathway but promote flowering when day length is lower than the critical value’, as described by Moutiq et al.. A synteny conservation approach based on comparative mapping between a maize genetic map and japonica rice physical map showed *LD* (*LUMINIDEPENDENS*) gene, an ortholog of the Arabidopsis gene involved in the autonomous pathway of flowering [39], associated with QTL for flowering time in bin 3.05 of maize chromosome 3 [24]. The response to environmental signals of the genes of this pathway has not clearly been established. Further research is required to determine the relationship between qDPS3 and *LD* gene.

In the present study, based on the data estimated in individual photoperiod environment, the QTL *qDPS10*, in the 10.04-region, was detected exclusively in long-day environment and had the greatest additive effects, accounting for 39.79%–44.30% of the phenotypic variance for DPS thermal time in Henan and Beijing long day environment. Further analysis indicated that *qDPS10* had the highest additive effect across environments and showed significant QE interaction effect both in short day environments and in long day environment, while opposite direction of additive×environment interaction effect were found in different photoperiod environments. This result suggested that *qDPS10* interact with both short-day and long-day environment and non-expression of *qDPS10* in short-day environment might result from opposite direction of additive and additive×environment interaction effect in Sanya. The decomposition of the total QTL effect allowed us to understand the genetic basis of flowering time and photoperiod sensitivity in maize more precisely. Therefore, the results of this study may provide valuable information for the identification and characterizing of genes responsible for PS. What' more, as *qDPS10*, most of QTL involved in QE interaction in this study do not exclusively response to certain photoperiod environment but respond to both short and long-day photoperiod environments and has opposite effects in different photoperiod environment.

In several previous studies, large-effect flowering time QTL [9], [14], [24], [26], [36], [40]–[42] and domestication traits [43] were also mapped to the vicinity of bin 10.04 on chromosome 10 in maize. Tian et al. report the discovery of a large region on 10.04 involved in adaptation or domestication that has been the target of strong selection during maize domestication [44]. Unlike previously described regions in the maize genome, 1.1 Mb and >15 genes lost genetic diversity during selection at this region. These studies suggest that 10.04-region may have played an important role in domestication and adaptation in maize. Further effort should be made to explore why 10.04-region had been under selection and which genes were involved in this important region.
